# Serum response factor promoting axonal regeneration by activating the Ras–Raf‐Cofilin signaling pathway after the spinal cord injury

**DOI:** 10.1111/cns.14585

**Published:** 2024-02-08

**Authors:** Limin Gao, Chen Zhang, Yonglin Zhu, Naili Zhang, Chunlei Zhang, Shuai Zhou, Guoying Feng, Fei Huang, Luping Zhang

**Affiliations:** ^1^ Institute of Neurobiology, Binzhou Medical University Yantai Shandong Province China; ^2^ Department of Neurobiology School of Basic Medical Sciences, Capital Medical University Beijing China; ^3^ Experimental Neurosurgery, Department of Neurosurgery Neuroscience Center, Frankfurt University Hospital Frankfurt am Main Germany; ^4^ Department of Bone and Joint Yantai Affiliated Hospital of Binzhou Medical University Yantai Shandong China; ^5^ University of Health and Rehabilitation Sciences Qingdao Shandong Province China

**Keywords:** axonal regeneration, Ras–Raf‐Cofilin, serum response factor, spinal cord injury

## Abstract

**Introduction:**

Serum response factor (SRF) is important in muscle development, tissue repair, and neuronal regulation.

**Objectives:**

This research aims to thoroughly examine the effects of SRF on spinal cord injury (SCI) and its ability to significantly impact the recovery and regeneration of neuronal axons.

**Methods:**

The researchers created rat models of SCI and scratch injury to primary spinal cord neurons to observe the expression of relevant factors after neuronal injury.

**Results:**

We found that the SRF, Ras, Raf, and cofilin levels increased after injury and gradually returned to normal levels. Afterward, researchers gave rats with SCI an SRF inhibitor (CCG1423) and studied the effects with nuclear magnetic resonance and transmission electron microscopy. The SRF inhibitor rodents had worse spinal cord recovery and axon regrowth than the control group. And the apoptosis of primary neurons after scratch injury was significantly higher in the SRF inhibitor group. Additionally, the researchers utilized lentiviral transfection to modify the SRF expression in neurons. SRF overexpression increased neuron migration while silencing SRF decreased it. Finally, Western blotting and RT‐PCR were conducted to examine the expression changes of related factors upon altering SRF expression. The results revealed SRF overexpression increased Ras, Raf, and cofilin expression. Silencing SRF decreased Ras, Raf, and Cofilin expression.

**Conclusion:**

Based on our research, the SRF promotes axonal regeneration by activating the “Ras–Raf‐Cofilin” signaling pathway.

## Graphical abstract

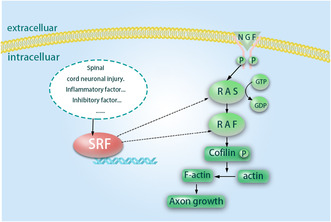

## 1 INTRODUCTION

Spinal cord injury (SCI) is a common paralytic disease in modern society, its incidence is increasing year by year. After SCI, the neuronal axis protrudes with different degrees of rupture, accompanied by inflammatory reactions and microenvironment changes. A series of complex pathophysiological changes affect the regeneration of neuronal axons.^1^ The regeneration ability of neurons in the central nervous system of adult mammals is extremely poor. Due to the lack of growth‐driving signals and subcellular mechanisms,^2^ if the axons of neurons cannot be compensated for regeneration, this will become an important reason for the difficult recovery after SCI. Therefore, how to effectively improve the intrinsic growth ability of neurons and improve the regeneration microenvironment of damaged neuron axons has become the key to the treatment of SCI.^3^

Serum response factor (SRF) is a highly conserved transcription Factor widely present in a variety of organisms. SRF can bind to a variety of genes, and 170 target genes have been found, including direct early genes (such as C‐FOS, fosB, junB, EGR‐1, and EGR‐2), neuronal genes (such as NURR1 and NUR77), and muscle genes (such as actins and myosins). By regulating the expression of these genes, SRF controls cell growth and differentiation, neuronal transmission, and muscle development and function.^4^ SRF also regulates cellular transcription through neurotrophic factors,^5^ neurotransmitters^6^ and factors that increase the intracellular calcium levels,^7^ stress factors, and viral activators.

At the same time, Henning Beck et al.^8^ believed that the signal axis of Factor (SRF) ‐Cofilin‐actin regulated the dynamics of mitochondria. The growth cone is composed of F‐actin and microtubules, and the protein of Actin polymerizes to F‐actin, which promotes the formation of the growth cone and thus provides power for the axon growth of neurons. In this process, the polymerization of actin protein is regulated by Cofilin, which is an actin‐depolymerizing factor. Phosphorylated cofilin promotes the formation of F‐actin, thus promoting axonal regeneration, which may also be an important target for SRF to play a role in nerve regeneration. Some nerve regeneration restraining factors such as: the source of oligodendrocytes Nogo, the source of astrocytes chondroitin sulfate proteoglycan (CSPG), is related with the SRF, can react by MAPK pathway mediated sign early genes regulating,^9^ and NGF–Ras–Raf–MAPK signaling pathway is an important way of promoting the NGF plays promote axon growth.^10^ Based on the above, we speculate that SRF plays an important role in the complex mechanism of promoting axon regeneration.

**FIGURE 1:**
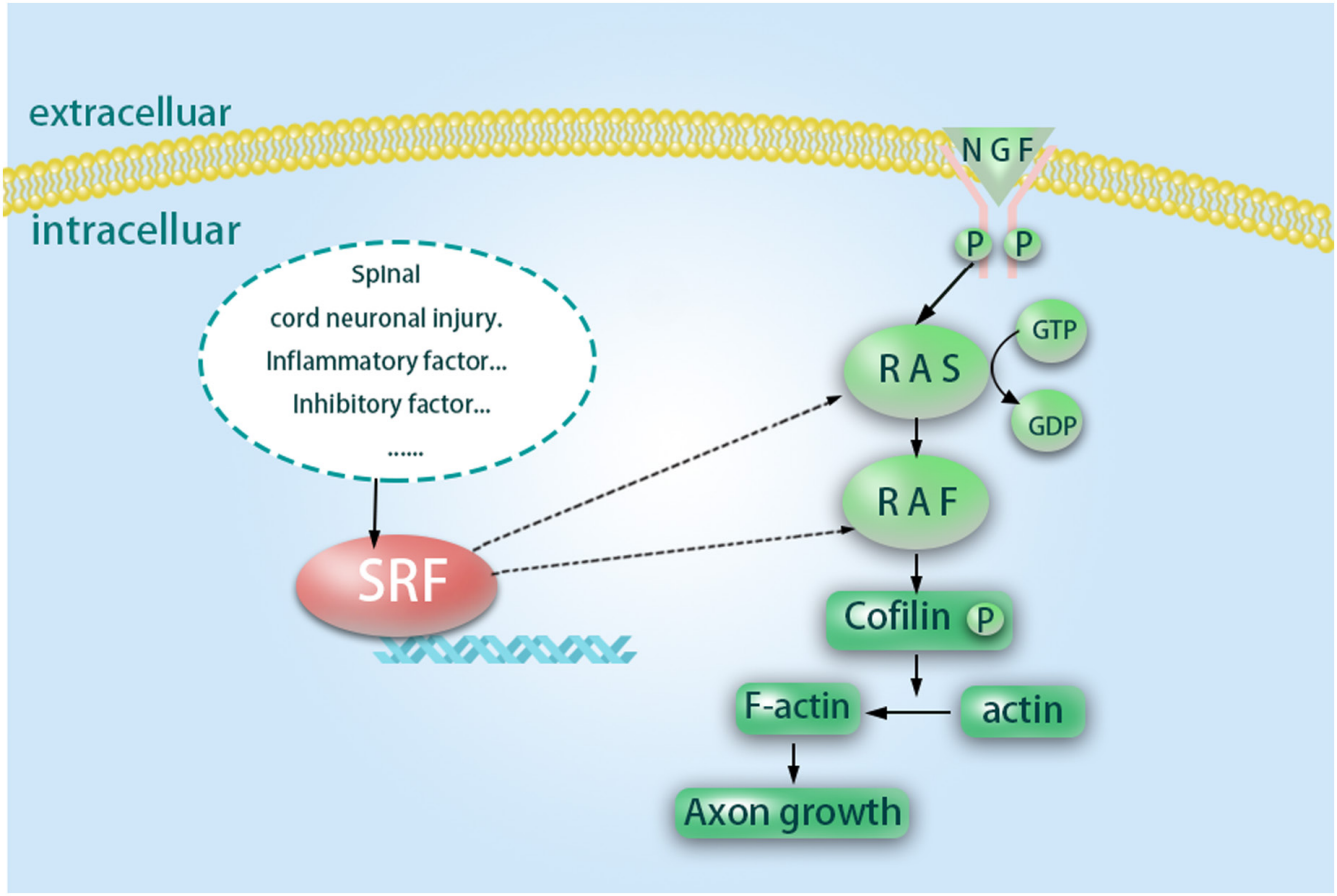
Serum response factor (SRF) mechanism pathway diagram.

In studies on facial nerve regeneration, the stimulation role of SRF in motor neuron survival^11^ and axon regeneration^12^ has been proved. However, the study of SRF in axonal regeneration after spinal cord neuron injury has not been reported. Our results showed that SRF expression was higher after SCI than in normal spinal cord, reached a peak on day 7 after injury, and tended to be normal on day 28 after injury, suggesting that SRF might be involved in the repair process of SCI. We speculated that SRF could regulate the cytoskeletal dynamics and promote axonal regeneration by regulating the gene transcription of actin and the activity of the mitotic protein. While adjusting cytoskeleton dynamics, SRF is crucial to the morphology and distribution of mitochondria, which is mainly involved in regulating mitochondrial transport, changing the microenvironment after nerve injury, and facilitating the regeneration of axons. The mechanism of action may first affect the activity of Ras or Raf, thus producing subsequent signal cascade reactions and finally regulating axonal regeneration through the pathway of “SRF–Ras–Raf–cofilin” (Figure 1).

## 2 RESULTS

### 2.1 SCI leads to alterations in the expression of SRF and its related proteins

**FIGURE 2:**
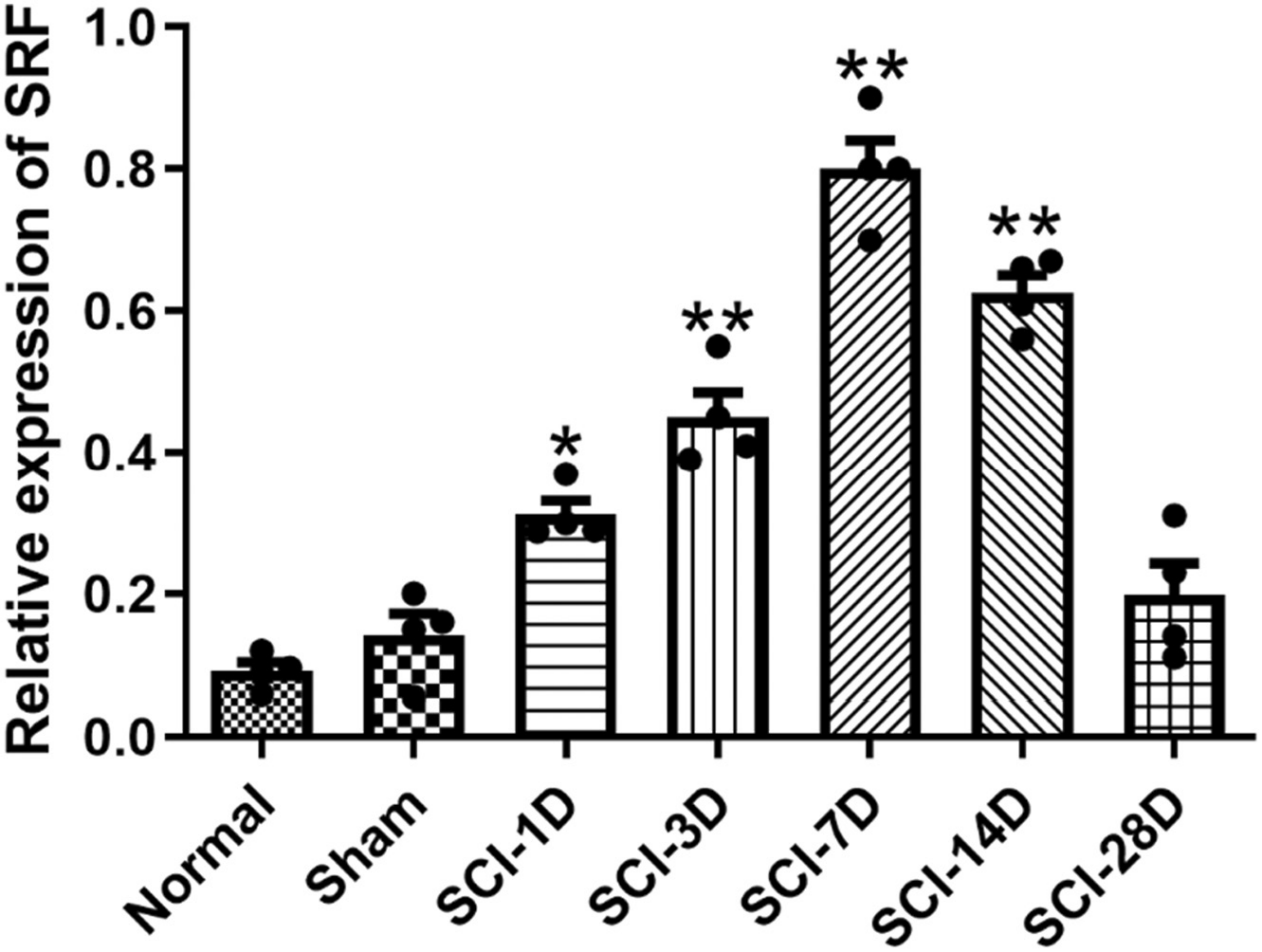
Expression of serum response factor (SRF) in serum of rats with spinal cord injury (SCI) at different time points (Data are presented as mean ± SD, *n* = 4 nerves per group. One‐way ANOVA with Tukey's multiple comparisons Test. *p < 0.05 and **p < 0.01).

In order to understand the expression levels of SRF at different time points after SCI, we performed Elisa detection on rat serum. The results showed expression of SRF was increased in the SCI group compared to the normal rats, reaching its peak on the 7th day after injury and returning to normal levels after 28 days (Figure 2).

**FIGURE 3:**
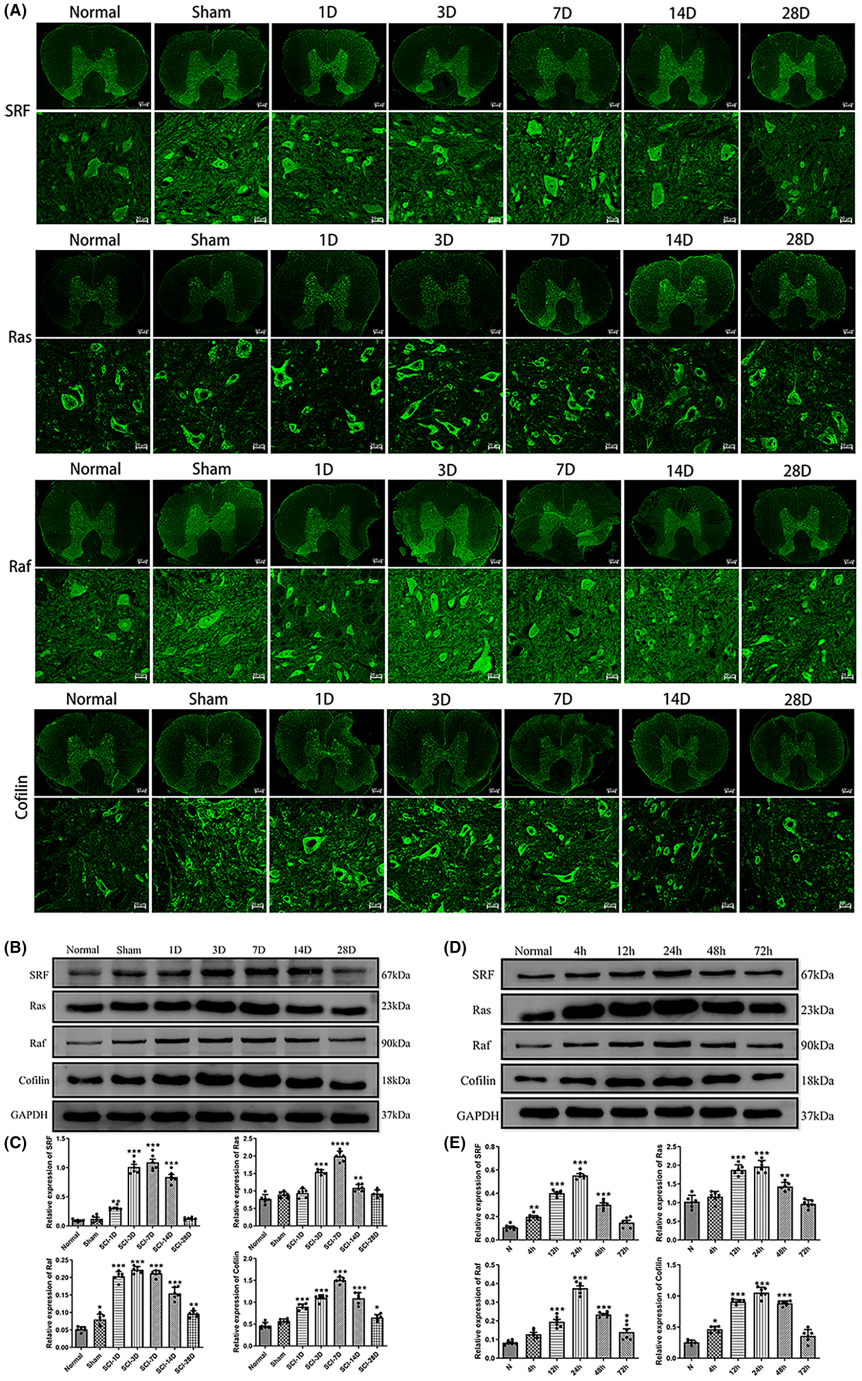
The expressions of different factors at different time points after spinal cord injury (SCI). (A) Immunofluorescence with the laser confocal microscope, Expression of serum response factor (SRF), Ras, Raf, cofilin, at different time points (1 day, 3 days, 7 days, 14 days, and 28 days) after SCI (the up scale bar = 200 μm, the down scale bar = 20 μm). (B, C) Western blot (WB) and quantitative results, experiments detected the expression changes of SRF, Ras, Raf, and Cofilin at different time points after SCI (Data are presented as mean ± SD, *n* = 6 nerves per group. One‐way ANOVA with Tukey's multiple comparisons Test. *p < 0.05; **p < 0.01;***p < 0.001; and ****p < 0.0001). (D, E) WB and quantitative results of the expression changes of SRF, Ras, Raf, and Cofilin at different time points after neuronal injury (Data are presented as mean ± SD, *n* = 6 nerves per group. One‐way ANOVA with Tukey's multiple comparisons test. *p < 0.05; **p < 0.01;***p < 0.001; ****p < 0.0001).

At a distance of about 0.5 cm from the center of the lesion, Through immunofluorescence experiments, we observed the expression of SRF, Ras, Raf, cofilin, and other factors at different time points (1 day, 3 days, 7 days, 14 days, and 28 days) after SCI. The experimental results demonstrated that these factors were predominantly expressed in the gray matter compared to the white matter of the spinal cord tissue. SRF and Raf was mainly expressed in the nucleus, while Ras, and cofilin, were primarily expressed in the cytoplasm (Figure 3A).

Subsequently, we used Western blot (WB) experiments to examine the expression changes of SRF, Ras, Raf, and Cofilin at different time points after SCI. The results revealed that SRF expression was higher in the SCI group than in the normal group, reaching its peak on the 7th day and returning to normal levels after 28 days. The Ras expression started to increase after SCI, reached its peak on the 7th day, and returned to normal levels on the 14th day. Raf expression was higher in the SCI group, peaked on the 3rd day, and remained elevated after 28 days. Cofilin expression was higher in the SCI group, reached its peak on the 7th day, and returned to normal levels after 28 days (Figure 3B,C).

We conducted similar validations at the cellular level by extracting primary spinal cord neurons and inducing injury through scratching. Using WB experiments, we investigated the expression changes of SRF, Ras, Raf, and Cofilin at different time points (4 h, 12 h, 24 h, 48 h, and 72 h) after neuronal injury. The experimental results showed that SRF expression was higher in the injured neurons (NIs) compared to the normal group, reaching its peak at 24 h and returning to normal levels after 72 h. Ras expression started to increase after neuronal injury, reached its peak at 24 h, and returned to normal levels at 48 h. The Raf's expression was higher than the normal group, peaked at 24 h, and remained elevated after 72 h. Cofilin expression was higher in the NIs, reached its peak at 24 h, and returned to normal levels after 72 h (Figure 3D,E).

### 2.2 Inhibition of the SRF expression impeded axon regeneration in rats following SCI

**FIGURE 4:**
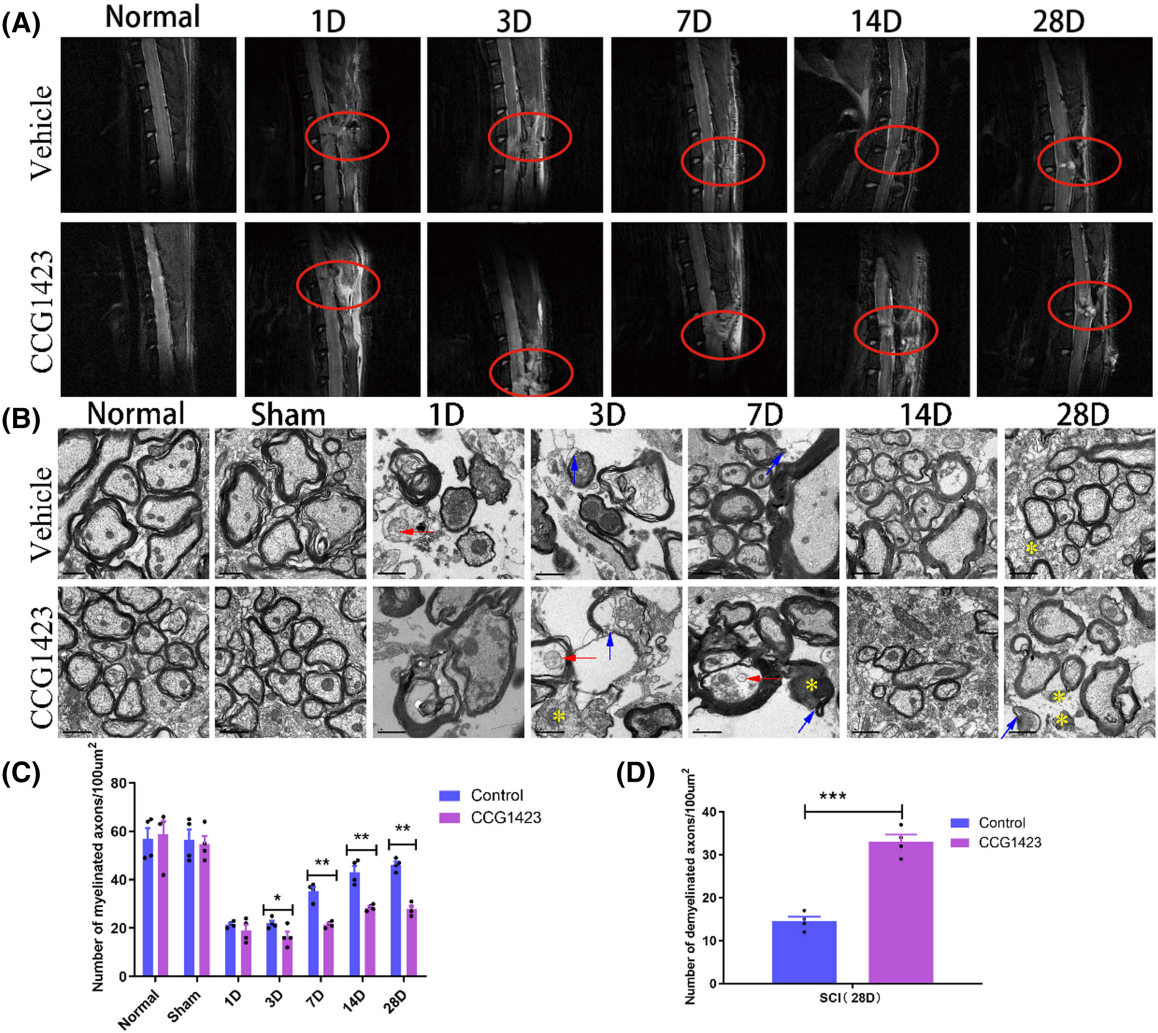
(A) MRI images at different time points after spinal cord injury (SCI), saline solution (vehicle group mean control group), SRF inhibitor group (CCG1423). The upper picture is the cross‐section of the SCI, the lower picture is the sagittal plane, and the circle is the injury site. (B) Transmission electron micrographs at different time points after the SCI. normal saline group (vehicle group = control group), SRF inhibitor group (CCG1423). Swollen mitochondria (red arrow); Loose, disintegrating myelin sheath (Blue arrow); shrunken, degenerated axons (Yellow asterisk). The length of the scale bar in the figure is 1 μm. (C) Statistical analysis of the number of myelinated axons at different time points after the SCI (Data are presented as mean ± SD, *n* = 4 brains per group. One‐way ANOVA with Tukey's multiple comparisons test, *p < 0.05; and **p < 0.01). (D) Statistical analysis of the number of demyelinated axons at 28 days after SCI (Data are presented as mean ± SD, *n* = 4 retinas per group. Unpaired *t* test, ***p < 0.001).

The recovery of SCI at different time points was observed by magnetic resonance imaging (MRI) after SRF inhibitor injection. The experimental results revealed that in the saline group, there was evident spinal cord disruption at the injury site on the first‐day post‐surgery, accompanied by significant muscle edema around the injury site. Over time, there was the growth of new axons and fusion of scar tissue at the site of spinal cord rupture, while the muscle edema gradually subsided. At 7 days post‐injury, fusion of the upper and lower ends of the spinal cord section could be observed, and by 28 days post‐injury, the upper and lower ends were essentially fused together. In contrast, the group treated with the SRF inhibitor (CCG1423) exhibited poor axon regeneration at the injury site, with no apparent fusion of the severed ends even at 14 days post injury. The recovery of SCI in the SRF inhibitor group was significantly inferior to that in the saline group (Figure 4A).

Transmission electron microscopy was used to observe the recovery of the spinal cord in rats following SCI after injection of an SRF inhibitor at different time points. The experimental results showed that in the saline group, there was evident myelin sheath disruption, axonal apoptosis, and mitochondrial dissolution in the spinal cord on the first‐day post‐injury. However, at 7 days post injury, dense myelin sheaths enveloping newly regenerated axons could be observed, and at 28 days post injury, a substantial number of newly regenerated axons were present. In the SRF inhibitor group (CCG1423), the quantity of newly regenerated axons was significantly lower than in the saline group, Along with more swollen mitochondria, atrophied axons, and myelin collapse (Figure 4B). We counted the number of axons in the SCI area at different time points, and found that the number of myelinated axons in the srf inhibitor group was significantly reduced (Figure 4C). At 28 days after injury, the number of demyelinated axons was significantly higher in the srf inhibitor group than in the control group (Figure 4D).

**FIGURE 5:**
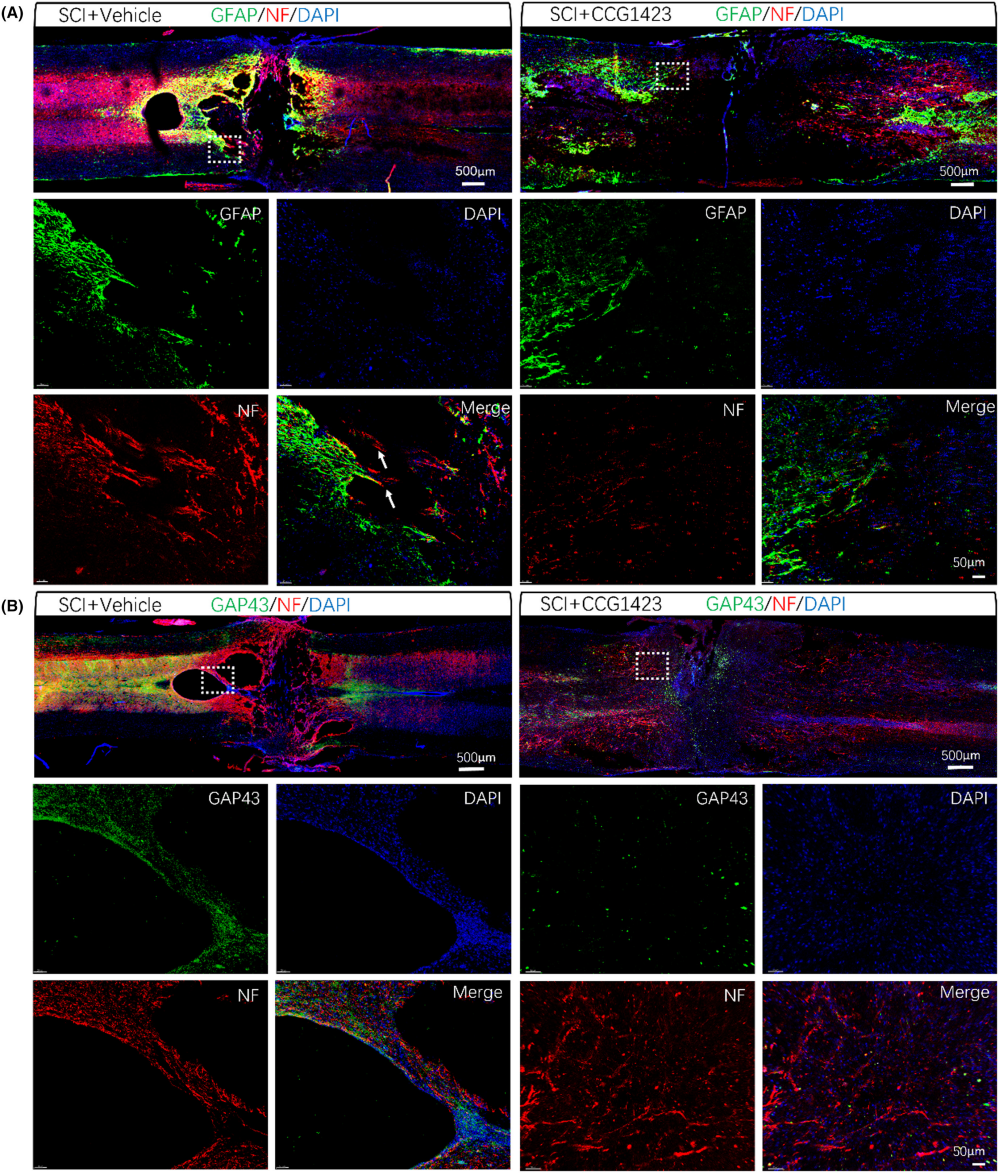
Immunofluorescence images of GFAP, GAP43, and NF. White arrows indicate axons crossing the glial scar (scale bar = 500 μm, scale bar = 50 μm).

Subsequently, immunofluorescence staining at the site of SCI was performed to further observe axonal growth. The results revealed that on day 28 post‐ SCI, neurofilament (NF) proteins were observed outside the glial scar in the injured area, but there is not enough evidence to determine whether these axons were newborn or surviving. However, due to the unfavorable microenvironment created by the SCI, these axons were unable to cross the injury site to establish synaptic connections (Figure 5A). Notably, in the group of spinal cord‐injured rats treated with the SRF inhibitor (CCG1423), the axons exhibited difficulty in crossing the glial scar. Additionally, we observed a significant decrease in GAP43 protein, which is closely associated with axonal regeneration and synaptic plasticity,^13^, ^14^ following the administration of CCG1423, This suggests that the axon's ability to regenerate is hampered. (Figure 5B).

Collectively, these findings suggest that the inhibition of SRF expression impedes axonal regeneration following SCI, indicating that SRF plays a protective role in promoting axonal regeneration.

### 2.3 Inhibiting the expression of SRF hindered the recovery of hind limb function in rats following the SCI

**FIGURE 6:**
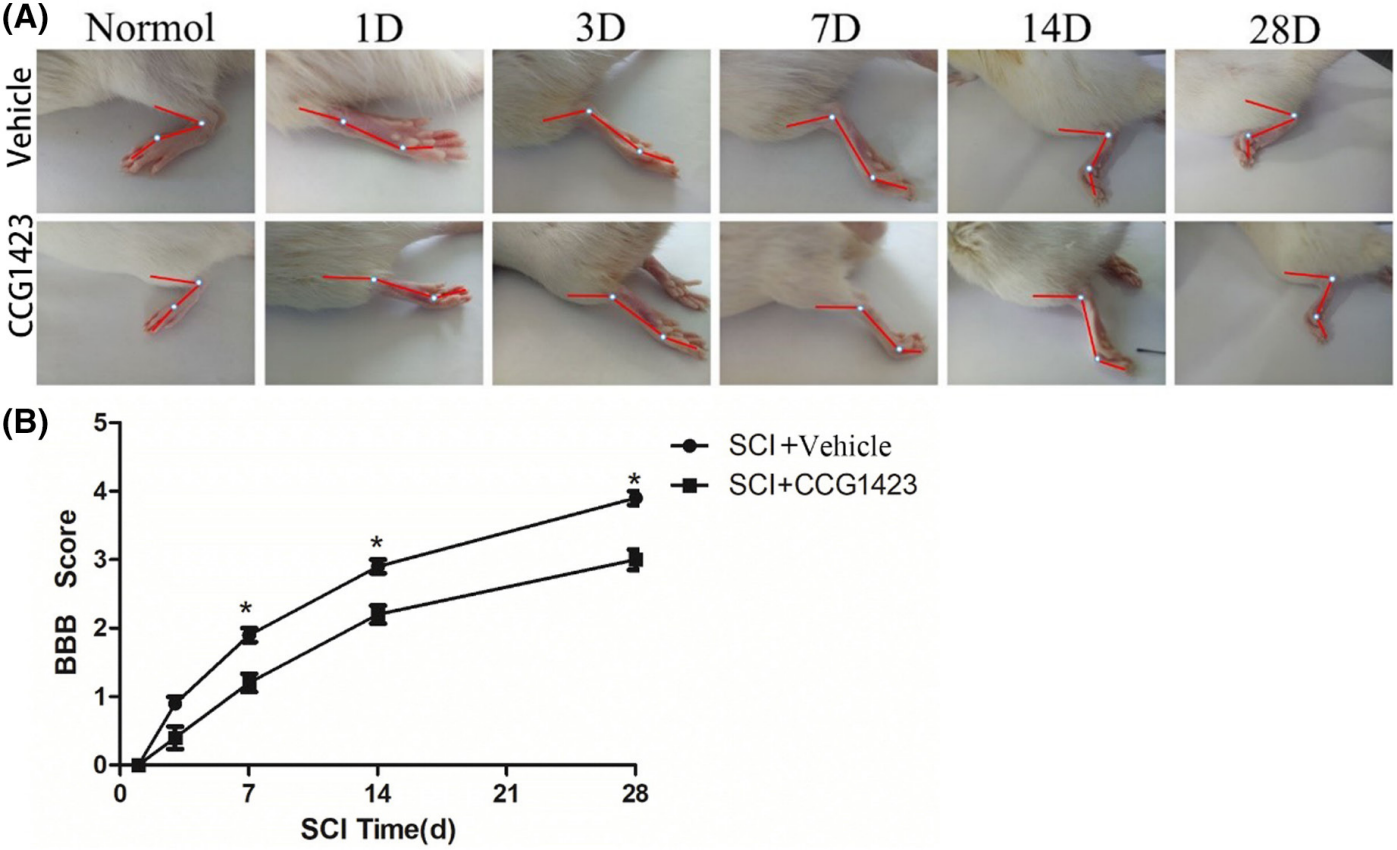
(A) Static images of hindlimb joints of rats with spinal cord injury (SCI) at different time points. (B) statistical analysis diagram of the BBB score (Data are presented as mean ± SD, *n* = 10 retinas per group. One‐way ANOVA with Tukey's multiple comparisons test, *p < 0.05. The recovery of the SCI + CCG1423 group at each time point after SCI was worse than that of the SCI + vehicle group.

The BBB functional score results showed that in the Normal group and Sham surgery group, rats exhibited sustained palm movement, continuous coordinated gait, persistent toe grip, claws remained parallel to the body during movement, stable trunk, and elevated tail, resulting in a BBB score of 21 points. At 1 day after complete spinal cord transection surgery, rats in the SCI group exhibited complete paralysis of the hind limbs, yielding a BBB score of 0 points. On the 3rd day after injury, slight movement was observed in one or two joints of the rats, resulting in a BBB score of 1 point. By the 7th day after injury, partial recovery of motor function was evident in the rats, with significant movement in one joint or substantial movement in one joint combined with slight movement in another joint, although the hind limbs could not support weight, yielding a BBB score of 2 points. At 14 days post‐injury, substantial movement was observed in both joints of the rats, leading to a BBB score of 3 points. By 28 days post‐injury, rats exhibited a degree of mobility in all three joints of the hind limbs, resulting in a BBB score of 4 points. Subsequent experiments revealed that rats injected with SRF inhibitor exhibited significantly inferior recovery compared to the control group. At 14 days post‐injury, the ability of the hind limbs and tail to move was still severely restricted in the SRF inhibitor‐treated group, with only two joints exhibiting slight movement. By 28 days post‐injury, rats in the SRF inhibitor‐treated group showed limited movement in two joints, but the hind limbs remained rigid. It is evident that functional recovery of spinal cord‐injured rats in the SRF inhibitor‐treated group was notably hindered (Figure 6).

### 2.4 Inhibiting the expression of SRF results in increased apoptosis of spinal cord neurons following injury

**FIGURE 7:**
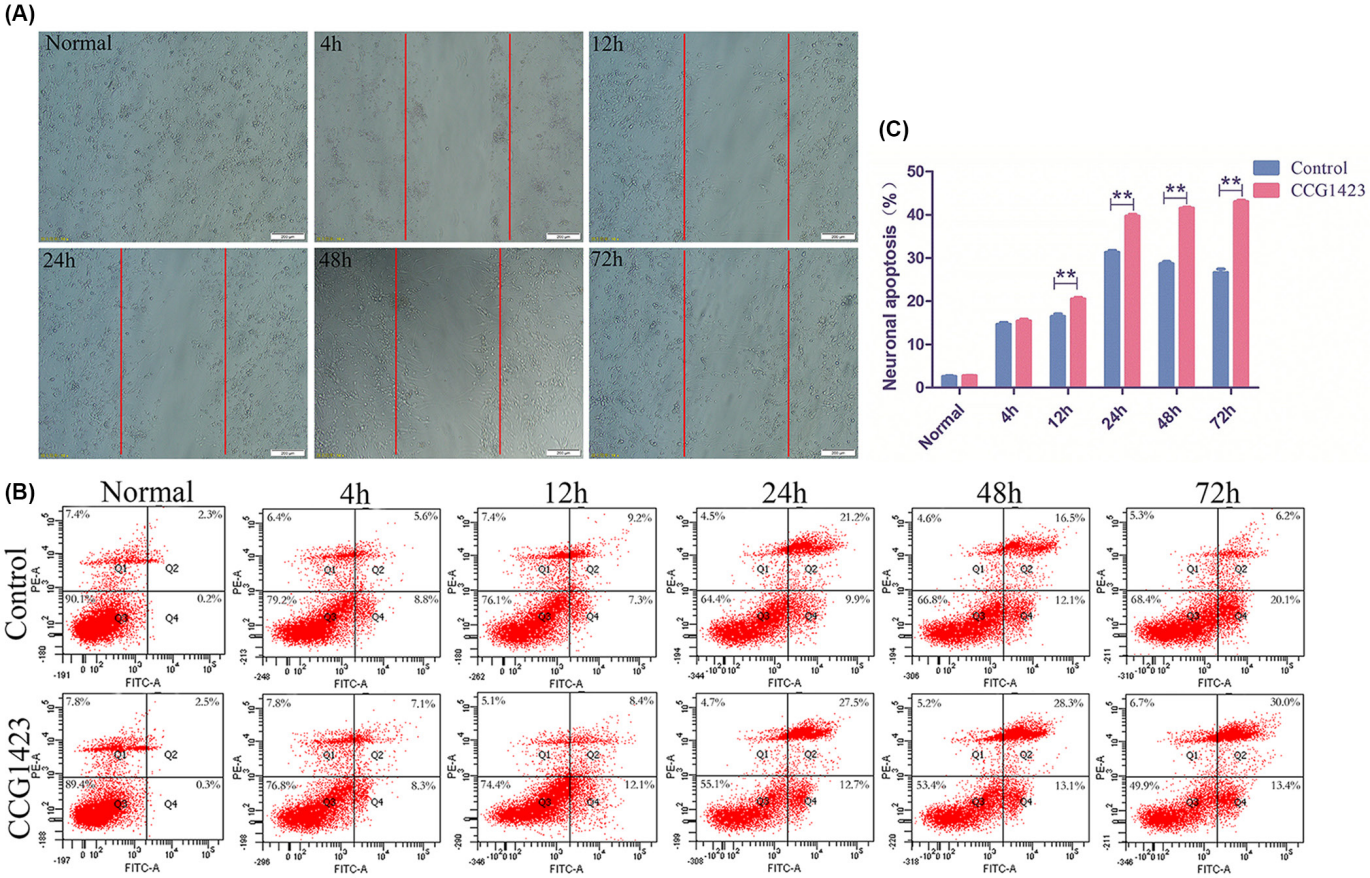
(A) The results of observations at different time points following scratch injury in primary neurons are presented in Figure A (magnification ×10, scale bar = 200 μm). (B, C) The apoptotic status at different time points following injury in primary neurons and the corresponding statistical analysisare presented (Data are presented as mean ± SD, *n* = 4 retinas per group. One‐way ANOVA with Tukey's multiple comparisons test, **p < 0.01). Physiological saline group (control group), SRF inhibitor group (CCG1423).

We performed scratch experiments to observe the axonal growth of neurons at different time points following neuronal scratch injury. The experimental results showed that with the passage of time, neuronal axons continuously extended toward the scratched area. At 24 h, new axonal extensions were already observed, and at 48 h, the axons on both sides of the scratch extended approximately one‐fourth of the width of the scratch. By 72 h, the axons on both sides of the scratch had largely fused together (Figure 7A).

To investigate the effects of an SRF inhibitor on primary neuronal cells at different time points following scratch injury, we conducted an apoptosis assay on spinal cord primary neurons at 4 h, 12 h, 24 h, 48 h, and 72 h post‐injury. The results revealed that in the control group without injury, the percentage of viable neurons was 90.1%, while the apoptotic cells accounted for 2.5%. At 4 h post‐injury, the percentage of viable neurons decreased to 79.2%, with an increase in apoptotic cells to 14.4%. At 12 h post‐injury, the percentage of viable neurons further decreased to 76.1%, and the apoptotic cells increased to 16.5%. At 24 h post‐injury, the percentage of viable neurons decreased to 64.4%, while the apoptotic cells increased to 31.1%. After 48 h, the number of apoptotic neurons slightly decreased to 28.6%. In the group treated with the SRF inhibitor, the percentage of viable neurons in the uninjured group was 89.4%, with apoptotic cells accounting for 2.8%. At 4 h post‐injury, the percentage of viable neurons decreased to 76.8%, and apoptotic cells increased to 15.4%. At 12 h post‐injury, the percentage of viable neurons further decreased to 74.4%, and apoptotic cells increased to 20.5%. At 24 h post‐injury, the percentage of viable neurons decreased to 55.1%, while apoptotic cells increased to 40.2%. After 48 h, the percentage of viable neurons decreased to 53.4%, and apoptotic cells increased to 41.4%. At 72 h post‐injury, the apoptotic rate continued to increase, with the percentage of viable neurons decreasing to 49.9% and apoptotic cells increasing to 43.4%. The results indicated that in the SRF inhibitor group, the apoptotic rate of neurons at 24 h, 48 h, and 72 h post‐injury was significantly higher than that in the control group (*p* < 0.01), and the apoptotic rate increased with prolonged time. Therefore, inhibiting the expression of SRF leads to increased apoptosis of spinal cord neurons following injury (Figure 7B,C).

### 2.5 Transfection of LV‐srf altered the migration capacity of damaged neurons

**FIGURE 8:**
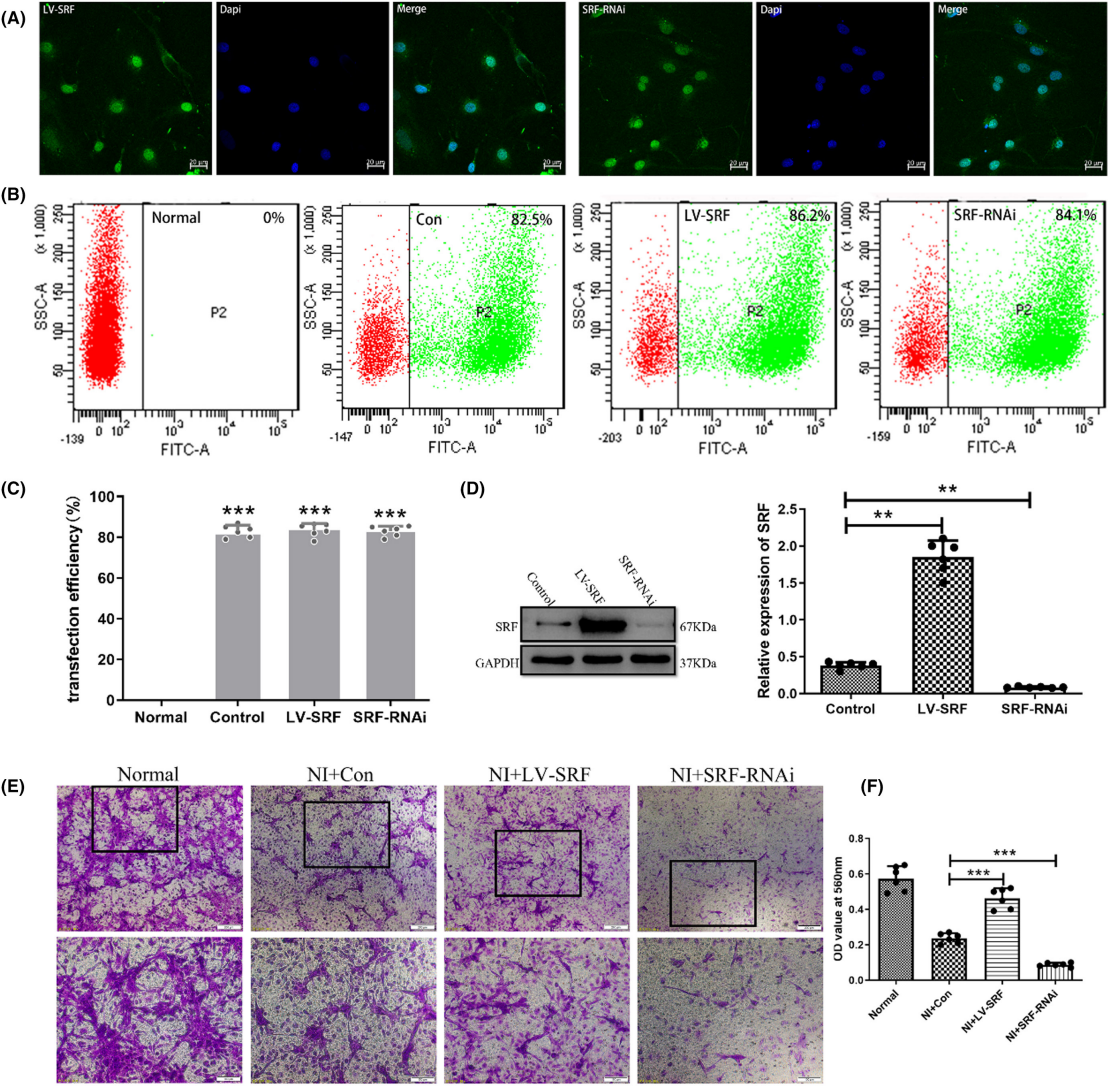
(A) Laser confocal microscopy was utilized to observe the virus's inherent green fluorescence. (B) Flow cytometry was employed to measure the viral transfection rate. (C) Statistical analysis plots were generated. (D) The relative expression level of the serum response factor (SRF) was determined through WB and quantitative analysis (Data are presented as mean ± SD, *n* = 6 retinas per group. One‐way ANOVA with Tukey's multiple comparisons test, **p < 0.01, ***p < 0.001). (E, F) control group (NI + Con), the group overexpressing SRF (NI + LV‐SRF) exhibited enhanced neuronal migration, while the SRF interference group (NI + SRF‐RNAi) showed weakened neuronal migration and quantitative analysis (Data are presented as mean ± SD, *n* = 6 retinas per group. One‐way ANOVA with Tukey's multiple comparisons test, ***p < 0.001.).

Based on preliminary experiments using lentiviral transduction, a multiplicity of infection (MOI) value of 100 was determined for neuronal transduction. The experimental results obtained from confocal microscopy and flow cytometry both showed transduction rates exceeding 80%. Following successful transduction, the relative expression level of SRF was assessed (Figure 8A–D).

To investigate the migratory capacity of neurons after injury and the impact of SRF on the migration of NIs, we used a Transwell chamber culture system. The results demonstrated that the migratory capacity of neurons after injury was significantly lower compared to normal neurons (Normal). In NI, the overexpression of SRF (NI + LV‐SRF) led to the increased neuronal survival and enhanced migration ability when compared to the empty vector control group (NI + Con). Conversely, interference with SRF expression (NI + SRF‐RNAi) resulted in reduced neuronal survival and weakened migratory capacity (Figure 8E,F).

### 2.6 The repair of spinal cord neuronal injury is affected by SRF through the Ras–Raf–cofilin pathway

**FIGURE 9:**
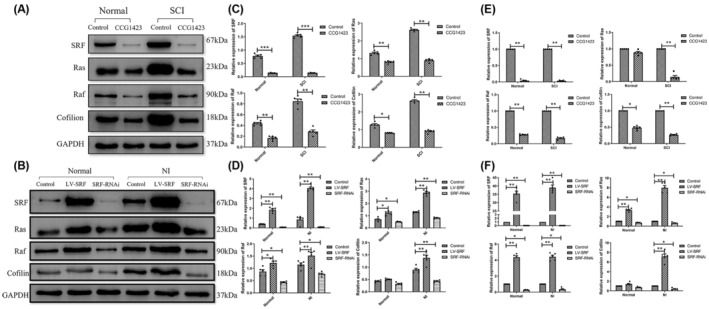
(A, C) Western blot (WB) results and statistical analysis of serum response factor (SRF)‐regulated factor expression in the spinal cord after injury (Data are presented as mean ± SD, *n* = 5 retinas per group. One‐way ANOVA with Tukey's multiple comparisons test, *p < 0.05, **p < 0.01, ***p < 0.001). (B, D) WB results and statistical analysis of SRF‐regulated factor expression in neurons after scratching injury (Data are presented as mean ± SD, *n* = 5 retinas per group. One‐way ANOVA with Tukey's multiple comparisons test, *p < 0.05, and **p < 0.01). (E) RT‐PCR results and the statistical analysis of SRF‐regulated factor expression in the spinal cord after injury. (F) RT‐PCR results and the statistical analysis of SRF‐regulated factor expression in neurons after scratching injury (Data are presented as mean ± SD, *n* = 5 retinas per group. One‐way ANOVA with Tukey's multiple comparisons test, *p < 0.05, **p < 0.01). CCG1423, SRF inhibitor; LV‐SRF, SRF overexpression; NI, Neuronal injury; SCI, spinal cord injury; SRF‐RNAi, SRF silencing.

The previous results have already demonstrated the expression changes of SRF, Ras, Raf, and Cofilin in spinal cord neurons after injury, indicating that SRF can protect axonal regeneration function. Moreover, the overexpression of SRF promotes the repair and migration of damaged neurons. To investigate the mechanism by which SRF exerts its function, we separately injected SRF inhibitors into normal rats and rats with SCI to observe the effects on Ras, Raf, and Cofilin expression. Experimental results showed that as SRF expression decreased, the expression of Ras, Raf, and Cofilin also decreased to varying degrees (Figure 9A). The impact of artificially inhibiting the SRF expression on Ras, Raf, and Cofilin was evaluated using RT‐PCR, and the results confirmed that decreased SRF expression led to a decrease in Ras, Raf, and Cofilin expression (Figure 9C).

Subsequently, lentiviral transduction was performed on normal neurons (Normal) and NI using different constructs (empty viral vector/control, SRF overexpression/LV‐SRF, SRF silencing/SRF‐RNAi) to observe the effects of modulating SRF expression on Ras, Raf, and Cofilin. The results showed that increasing the SRF expression led to an increase in the Ras, Raf, and Cofilin expressions to varying degrees, while decreasing the SRF expression resulted in a decrease in Ras, Raf, and Cofilin expression (Figure 9B). These findings were further confirmed by RT‐PCR analysis, demonstrating that higher SRF expression correlated with increased expression of Ras, Raf, and Cofilin, whereas lower SRF expression led to a decreased expression of these factors (Figure 9D).

Taken together, these results indicate that SRF directly interacts with Ras and Raf to regulate Cofilin expression, thereby promoting axonal regeneration and exerting neuroprotective effects through the “Ras–Raf‐Cofilin” signaling pathway.

## 3 DISCUSSION

Spinal cord injury can lead to severe and irreversible functional loss. The lack of regenerative capacity in neurons themselves, as well as changes in the microenvironment following injury, are obstacles to SCI repair. Currently, the main treatments for SCI include neuroprotective drugs,^15^ stem cell transplantation,^16^ bioactive materials,^17^ and AAV‐mediated transdifferentiation of glial cells.^18^, ^19^ In general, these treatments provide a favorable microenvironment for reconstructing damaged neural circuits and promoting functional recovery. Promoting axonal regeneration remains a key aspect of SCI repair.

The SRFis a transcription factor that regulates genes involved in various cellular activities, such as proliferation, migration, differentiation, angiogenesis, and apoptosis.^20^, ^21^ In this study, we investigated the expression of SRF at different time points after spinal cord neuron injury in both animal experiments and cell experiments. We explored the role of SRF in neuronal injury repair and investigated the mechanisms by which SRF promotes axonal regeneration after SCI. The experimental results showed an upregulation of SRF expression after SCI, reaching a peak and gradually returning to normal levels as the injury progressed. On the 28th day after injury, scar tissue around the injury site was observed by MRI, and the damaged ends of the spinal cord were found to have reconnected. This suggests the existence of a self‐repair process in spinal cord‐injured rats, but this self‐repair process is far from sufficient to restore normal motor function. Injection of an SRF inhibitor resulted in significantly poorer alignment of the spinal cord ends in rats compared to the control group. Transmission electron microscopy showed a reduced number of myelin sheaths around the axons, and the expression of growth‐associated protein 43 (GAP43) related to axonal regeneration^22^ was inhibited, indicating that the absence of SRF inhibited SCI recovery and impeded axonal regeneration. We found that neuronal injury caused apoptosis, and the artificial intervention resulting in the loss of SRF further aggravated the process of apoptosis, indicating the neuroprotective role of SRF.

Primary neurons were transfected with lentiviral vectors to overexpress or silence SRF. The results showed enhanced migration ability in neurons with SRF overexpression and weakened migration ability in the SRF‐silenced group. Similarly, studies have shown that the loss of SRF leads to ectopic aggregation of neurons in the subventricular zone (SVZ) of the brain,^9^ which is a major neurogenic region in the brain. The migration of SVZ cells lacking SRF toward the olfactory bulb was impaired. Additionally, it has been shown that SRF guides the migration of pericytes downstream of PDGFRB signaling and mediates pathological pericyte activation during ischemic retinopathy.^23^ Cell migration involves complex interactions between the extracellular matrix and the intracellular actin cytoskeleton. Therefore, the actin cytoskeleton can be considered a key link between the extracellular environment and intracellular signaling pathways. Many SRF target genes encode components of the actin cytoskeleton, so the structure and dynamics of the actin cytoskeleton are disrupted in cells and tissues lacking SRF. Cofilin is an actin‐binding protein that is typically involved in the dynamic turnover of actin filaments. Dephosphorylated cofilin binds to ADP‐actin and forms a rod‐like structure known as cofilin‐actin,^24^ which halts actin‐dependent processes locally. Ras is a small GTP‐binding protein that acts as a molecular switch on the cell membrane, initiating signaling pathways that promote cell growth and proliferation.^25^ Raf protein kinase is a major effector of Ras. The specific assembly of Ras–Raf is further extended on the membrane by the dimerization of mitogen‐activated protein kinase (MAPK), Ras kinase inhibitor dimers, and extracellular signal‐regulated kinase dimers, ultimately leading to the activation of cofilin in the cytoplasm.^26^ Meanwhile, Henning Beck and others have suggested that the factor SRF‐cofilin‐actin axis regulates mitochondrial dynamics. To further investigate whether SRF directly regulates the expression of Ras, Raf, and cofilin, this study used lentiviral vectors to overexpress or silence the SRF gene and assessed the expression levels of Ras, Raf, and cofilin. We found that the expression levels of Ras, Raf, and cofilin increased with elevated SRF levels and decreased with decreased SRF levels, indicating a consistent relationship between their expression levels and SRF. Therefore, we believe that SRF can directly interact with Ras, Raf, and cofilin and promote axonal regeneration and neuroprotection through the “Ras–Raf‐cofilin” signaling pathway.

This study analyzed the role of SRF in spinal cord neuron injury from both in vitro and in vivo perspectives, investigating whether SRF exerts its effects on axonal regeneration of neurons through the “Ras–Raf‐cofilin” pathway. By exploring new mechanisms and targets for neuronal axonal regeneration and repair, this study provides experimental evidence and new insights for the development of drugs related to SCI.

## 4 METHODS

### 4.1 Experimental animals

Experimental animals were SD rats, which were grouped according to different time points after SCI. Experimental animals were divided into a normal group, sham group, 1D group, 3D group, 7D group, 14D group, and 28D group.

### 4.2 The SCI model

Female SD rats were purchased and bred for 1 week under standard conditions. In an aseptic operation, the skin and superficial fascia were incised with t9‐T10 segment osseous marks as the center. Blunt dissection of paravertebral muscles on both sides of the spinous process was performed to expose the vertebral lamina, the vertebral lamina was bitten to expose the spinal cord, and the spinal cord was transected with a fiber shear (in the sham operation group, only the vertebral lamina was bitten but the spinal cord was not injured). At this time, the tension of the lower limbs and tail disappeared completely, and the arch of the foot was reversed. Suture the muscle, fascia, and skin layer by layer. Three to four rats in a cage were kept warm and kept constant temperature. Artificial bladder massage was performed every day, once in the morning and once in the evening.^27^, ^28^, ^29^

### 4.3 Functional analysis

According to Basso, Beattie, and Brenham (BBB) standards, hindlimb etiology of rats was independently observed on 1D, 3D, 7D, 14D, and 28D after SCI, respectively, and activity levels of ankle, knee, and hip joints were recorded. Two experimenters independently evaluated and scored according to THE BBB scoring criteria, and then took the mean value, and statistically analyzed the changes of the BBB score of rats.

### 4.4 SRF inhibitor model preparation

Preparation of rat SCI model was conducted. The inhibitor of SRF, CCG1423, was dissolved in dimethyl sulfoxide (DMSO), and the working solution was diluted with physiological saline. The freshly prepared solution was administered via intraperitoneal injection to the injury group at a dosage of 0.15 mg/kg per day (0.15 mg/kg; i.p. once daily). The control group received intraperitoneal injections of physiological saline containing an equivalent amount of DMSO.

### 4.5 Magnetic resonance imaging

Rats in the SRF inhibitor group and normal saline group were sent to the small animal nuclear magnetic laboratory of Binzhou Medical College. The rats were fixed with body coils, and the NMR parameters were set. The changes of the rats at different time points after SCI were observed from cross‐section and sagittal plane respectively.

### 4.6 Transmission electron microscopy

After 4% paraformaldehyde was instilled, 0.5 mm^3^ of the spinal tissue was taken and soaked for 2 h with 2.5% glutaraldehyde. The samples were sent to the electron microscope room of Binzhou Medical College, washed three times with phosphate‐buffered saline (PBS), and soaked overnight in PBS. At 4°C, the samples were fixed with 1% osmium acid for 90 min, and the samples were shaken every 20 min. PBS was washed three times and the gradient alcohol was dehydrated. Acetone was passed twice, 10 min each. The samples were soaked in an acetone and resin mixture of the same amount, vacuumized for 10 min, and soaked overnight. The samples were heated and polymerized to embed the tissue. The apoptosis of neuronal axons and myelin sheath was observed by electron microscope after the ultrathin section.

### 4.7 Immunofluorescence

Spinal cord tissue, or neurons were fixed in 4% PFA for 20 min. Thenwe were incubated with primary rabbit anti‐SRF antibody (1:200 diluted by PBS, CST, D71A9, USA), anti‐Ras antibody (1:500 diluted by PBS, Abcam, ab52939, USA), anti‐Raf antibody (1:250 diluted by PBS, Abcam, ab33899, USA), anti‐cofilin antibody (1:300 diluted by PBS, Abcam, ab42824, USA), anti‐GAP43 antibody (1:500 diluted by PBS, Abcam, ab75810, USA) and anti‐NF antibody (1:50 diluted by PBS, ZSGB‐BIO, 18703E05, China) overnight at 4°C, incubator with DAPI (1:700 diluted by PBS, Solarbio, China) 10 min, and washed with PBS for 5 times (3 min each time). After washing, the secondary antibody (1:500, Abcam, USA) was applied, and the mixture was incubated at 37°C for 1 h. Images were recorded using a microscope.

### 4.8 Western blot

Spinal cord tissue or neurons lysed in RIPA lysis buffer containing protease and phosphatase inhibitors. After centrifugation at 12,000 r for 10 min at 4°C, the cell fragments were removed and the supernatant proteins were extracted. Protein concentration (BCA protein concentration determination kit, Beyotime, China) was detected. Gel was prepared (SDS–PAGE gel preparation kit, Beyotime, China). The upper sample quantity of each group was 30 μg. Then electrophoretic fluid was added, and electrophoresis was started (concentration of adhesive voltage constant 80 V, 30 min; separation of the adhesive voltage constant 100 V, 90 min). Tailor the target protein according to the color band position of the mark protein. The protein was transferred to the PVDF membrane. After being blocked with nonfat dry milk, the membranes were incubated with primary rabbit anti‐SRF antibody (1:1000 diluted by PBS, CST, D71A9, USA), anti‐Ras antibody (1:2000 diluted by PBS, Abcam, ab52939, USA), anti‐Raf antibody (1:2000 diluted by PBS, Abcam, ab33899, USA), anti‐cofilin antibody (1:1000 diluted by PBS, Abcam, ab42824, USA), and anti‐GAPDH antibody (1:1000 diluted by TBST, CST, #2118, USA) overnight at 4°C. The membranes were then incubated with the secondary antibody (1:5000, diluted by TBST, USA) at room temperature for 2 h, after three rinses. The blot signal was detected using an ECL detection kit (Millipore, USA) and analyzed with Image J software.

### 4.9 RT‐PCR

Total RNA was extracted from spinal cord tissue and neurons using the RNAiso Plus extraction kit (Takara). For RT‐PCR, Primers of Ras, Raf, Cofilin, and SRF were synthesized to produce 100–150 bp fragments (Table S1). GAPDH was conducted as a control. Then, 1 μg of total RNA was subjected to cDNA synthesis using a reverse‐transcription kit (Takara). cDNA synthesis and amplification were conducted in a Bio‐Rad PCR system. The conditions for real‐time PCR were as follows: 95°C for 10 s and 60°C for 45 s, for a total Mol Neurobiol for 40 cycles. The results were analyzed by the relative quantification method.

### 4.10 Primary neuron extraction and identification

The female SD rats were selected to be 18 days pregnant. The uterus was removed by laparotomy, the placenta was removed, and the spinal cord of the fetal rat was quickly separated and temporarily stored in a 0°C Dulbecco's Modified Eagle's Medium (DMEM) (Gibco, USA). The spinal blood vessel and the membrane were removed under the stereomicroscope. The spinal cord was put into a 15 mL centrifuge tube in which Trypsin (Gibco, USA) was added for digestion at 37°C for 15 min. DMEM‐containing serum was then added to stop digestion. The cells were filtered using a 200 mesh cell screen and centrifuged at 1200 rpm for 5 min. The supernatant was removed and the planting medium (DMEM + 20% FBS, Gibco, USA) was added again. After cell counting, the cells were inoculated into polylysine‐coated culture plates at a density of 106 cells/mL, and cultured at 37°C with 5% CO_2_. Four hours later, the medium was replaced with a special culture medium for neuron growth (neurobasal+B27, Gibco, USA). Cytarabine (2.5 μg/mL) was added to the medium, and half of the solution was replaced with culture for 3 days to inhibit the growth of glial cells and obtain the purest primary neurons. The neurons were cultured until the seventh day, and identified by immunofluorescence assay with specific mark antibody β3‐tubulin.

### 4.11 Preparation of the neuronal injury model

The primary neurons were extracted and cultured on the seventh day. The culture medium was sucked off, washed twice with PBS, and the back of the sterile surgical blade was used to make a “#” scratch, causing mechanical damage. Scratch damage was performed on a pre‐designed standard template, which was marked with thin lines parallel to horizontal and vertical lines, and the parallel spacing between the lines was set to 0.5 cm. The template was laid under the culture plate to control the strength and speed of the scratch. Under direct vision, the sterile surgical blade was used to cut along the line of the template to damage the neurons and make the neuron scratch damage model.^30^, ^31^

### 4.12 Detection of the apoptosis rate by flow cytometry

At 4 h, 12 h, 24 h, 48 h, and 72 h after damage, neurons were digested with trypsin and centrifuged for collection. The cells were separated into 0.5 × 10^5^ ~ 5 × 10^5^ per tube, then centrifuged at 1200 r for 5 min, washed with PBS, and centrifuged again. AnnexinV‐FITC and PI were added to stain cells and mixed gently. The samples without AnnexinV‐FITC and PI were set as the negative control, only AnnexinV‐FITC and only PI were added as adjustment compensation. All samples underwent incubation without light exposure for 10 ~ 15 min, and then flow cytometry.

### 4.13 Lentivirus transfection

The primary neurons were seeded in 6‐well plates, and 10^6^ cells were cultured in each well until the 7th day. Lentivirus transfection was performed when the cells were observed to be in good and stable condition. cultured primary neurons were divided into three groups: negative control virus, SRF overexpressed virus (LV‐SRF, designed by Takara), and SRF silencing virus (SRF‐RNAi, designed by Takara). According to the calculation formula: virus volume = (MOI × number of cells)/virus titer, the required virus volume for each well was calculated. Add the virus, and add the reagent to help the virus transfection, culture at 37°C for 12 h, change the medium, and continue the culture. Infection efficiency was observed about 72 h after infection.

### 4.14 Neuronal migration experiment

Model neuronal injury was prepared. The cells were digested and centrifuged, and the cells were resuspended with DMEM without serum and counted. A volume of 100 μL cell suspension is added to the upper chamber, with a cell density of 10^6^/mL, and 600 μL neuron culture medium is added to the lower chamber (98% neuronal +2%B27). After being cultured at 37°C for 18 h, the Transwell chamber was taken out, and cells near the inner side of the PVPF membrane were wiped off with a cotton swab. The outer cells were fixed with 4% paraformaldehyde for 20 min, stained with 0.1% crystal violet for 10 min, and washed three times with water to remove the excess dye. The Transwell chamber was placed on a clean slide and an inverted microscope was used to observe the number of cells in the outer layer. At the same time, crystal violet was eluted with 33% acetic acid solution and the absorbance was read at 560 nm.

### 4.15 Statistical analyses

All data were statistically analyzed and plotted using GraphPad Prism 7 software and are expressed as the mean ± SD. Shapiro–Wilk test was used to test the data normality. Data that do not exhibit a normal/Gaussian distribution are analyzed by nonparametric equivalence. Results between the two groups were performed using an unpaired *t*‐test. The results of the same treatment performed at different time points were compared with one‐way ANOVA.

## AUTHOR CONTRIBUTIONS

LG, CZ, and YZ: made substantial contributions to the conception or design of the work; LG and CZ: responsible for the acquisition, analysis, or interpretation of data for the work; ClZ, SZ, GF, and NZ: took part in drafting or revising the paper; LZ and FH: responsible for the final approval of the version to be published.

## FUNDING INFORMATION

We acknowledge the work was supported by the following grants: the National Natural Science Fund of China (31700930 and 81870985).

## CONFLICT OF INTEREST STATEMENT

The authors declare no competing interests.

## Supporting information


Table S1.

Data S1.

## Data Availability

All relevant data are available from the corresponding author upon any reasonable request. Source data are provided with this paper.

## References

[1] Parlakian A , Tuil D , Hamard G , et al. Targeted inactivation of serum response factor in the developing heart results in myocardial defects and embryonic lethality. Mol Cell Biol. 2004;24(12):5281‐5289. doi:10.1128/MCB.24.12.5281-5289.2004 15169892 PMC419888

[2] Niu Z , Iyer D , Conway SJ , et al. Serum response factor orchestrates nascent sarcomerogenesis and silences the biomineralization gene program in the heart. Proc Natl Acad Sci U S A. 2008;105(46):17824‐17829. doi:10.1073/pnas.0805491105 19004760 PMC2584699

[3] Miano JM , Long X , Fujiwara K . Serum response factor: master regulator of the actin cytoskeleton and contractile apparatus. Am J Physiol Cell Physiol. 2007;292(1):C70‐C81. doi:10.1152/ajpcell.00386.2006 16928770

[4] Bai X , Mangum KD , Dee RA , et al. Blood pressure‐associated polymorphism controls ARHGAP42 expression via serum response factor DNA binding. J Clin Invest. 2017;127(2):670‐680. doi:10.1172/JCI88899 28112683 PMC5272192

[5] Erratum: reduced nuclear translocation of serum response factor is associated with skeletal muscle atrophy in a cigarette smoke‐induced mouse model of COPD [Corrigendum]. Int J Chron Obstruct Pulmon Dis. 2018;13:603. doi:10.2147/COPD.S157992 29497288 PMC5819583

[6] Filomena MC , Yamamoto DL , Caremani M , et al. Myopalladin promotes muscle growth through modulation of the serum response factor pathway. J Cachexia Sarcopenia Muscle. 2020;11(1):169‐194. doi:10.1002/jcsm.12486 31647200 PMC7015241

[7] Sakuma K , Nakao R , Inashima S , Hirata M , Kubo T , Yasuhara M . Marked reduction of focal adhesion kinase, serum response factor and myocyte enhancer factor 2C, but increase in RhoA and myostatin in the hindlimb dy mouse muscles. Acta Neuropathol. 2004;108(3):241‐249. doi:10.1007/s00401-004-0884-5 15221330

[8] Kong M , Chen X , Lv F , et al. Serum response factor (SRF) promotes ROS generation and hepatic stellate cell activation by epigenetically stimulating NCF1/2 transcription. Redox Biol. 2019;26:101302. doi:10.1016/j.redox.2019.101302 31442911 PMC6831835

[9] Alberti S , Krause SM , Kretz O , et al. Neuronal migration in the murine rostral migratory stream requires serum response factor. Proc Natl Acad Sci U S A. 2005;102(17):6148‐6153. doi:10.1073/pnas.0501191102 15837932 PMC1087932

[10] Stritt C , Stern S , Harting K , et al. Paracrine control of oligodendrocyte differentiation by SRF‐directed neuronal gene expression. Nat Neurosci. 2009;12(4):418‐427. doi:10.1038/nn.2280 19270689

[11] Miano JM . Role of serum response factor in the pathogenesis of disease. Lab Invest. 2010;90(9):1274‐1284. doi:10.1038/labinvest.2010.104 20498652

[12] Stritt C , Knöll B . Serum response factor regulates hippocampal lamination and dendrite development and is connected with reelin signaling. Mol Cell Biol. 2010;30(7):1828‐1837. doi:10.1128/MCB.01434-09 20123976 PMC2838085

[13] Spencer S , Willard MB . Does GAP‐43 support axon growth by increasing the axonal transport velocity of calmodulin? Exp Neurol. 1992;115(1):167‐172. doi:10.1016/0014-4886(92)90243-j 1370220

[14] Tedeschi A , Nguyen T , Puttagunta R , Gaub P , Di Giovanni S . A p53‐CBP/p300 transcription module is required for GAP‐43 expression, axon outgrowth, and regeneration. Cell Death Differ. 2009;16(4):543‐554. doi:10.1038/cdd.2008.175 19057620

[15] Wu C , Chen H , Zhuang R , et al. Betulinic acid inhibits pyroptosis in spinal cord injury by augmenting autophagy via the AMPK‐mTOR‐TFEB signaling pathway. Int J Biol Sci. 2021;17(4):1138‐1152. doi:10.7150/ijbs.57825 33867836 PMC8040310

[16] Gao L , Peng Y , Xu W , et al. Progress in stem cell therapy for spinal cord injury. Stem Cells Int. 2020;2020:2853650. doi:10.1155/2020/2853650 33204276 PMC7661146

[17] Wang Z , Duan H , Hao F , et al. Circuit reconstruction of newborn neurons after spinal cord injury in adult rats via an NT3‐chitosan scaffold. Prog Neurobiol. 2023;220:102375. doi:10.1016/j.pneurobio.2022.102375 36410665

[18] Zhou H , Su J , Hu X , et al. Glia‐to‐neuron conversion by CRISPR‐CasRx alleviates symptoms of neurological disease in mice. Cell. 2020;181(3):590‐603.e16. doi:10.1016/j.cell.2020.03.024 32272060

[19] Li Y , He X , Kawaguchi R , et al. Microglia‐organized scar‐free spinal cord repair in neonatal mice. Nature. 2020;587(7835):613‐618. doi:10.1038/s41586-020-2795-6 33029008 PMC7704837

[20] Miranda MZ , Lichner Z , Szászi K , Kapus A . MRTF: basic biology and role in kidney disease. Int J Mol Sci. 2021;22(11):6040. doi:10.3390/ijms22116040 34204945 PMC8199744

[21] Ni H , Haemmig S , Deng Y , et al. A smooth muscle cell‐enriched Long noncoding RNA regulates cell plasticity and atherosclerosis by interacting with serum response factor. Arterioscler Thromb Vasc Biol. 2021;41(9):2399‐2416. doi:10.1161/ATVBAHA.120.315911 34289702 PMC8387455

[22] Lan G , Cai Y , Li A , Liu Z , Ma S , Guo T . Association of presynaptic loss with Alzheimer's disease and cognitive decline. Ann Neurol. 2022;92(6):1001‐1015. doi:10.1002/ana.26492 36056679

[23] Orlich MM , Diéguez‐Hurtado R , Muehlfriedel R , et al. Mural cell SRF controls pericyte migration, vessel patterning and blood flow. Circ Res. 2022;131(4):308‐327. doi:10.1161/CIRCRESAHA.122.321109 35862101 PMC9348820

[24] Bamburg JR , Minamide LS , Wiggan O , Tahtamouni LH , Kuhn TB . Cofilin and actin dynamics: multiple modes of regulation and their impacts in neuronal development and degeneration. Cell. 2021;10(10):2726. doi:10.3390/cells10102726 PMC8534876 34685706

[25] Chen K , Zhang Y , Qian L , Wang P . Emerging strategies to target RAS signaling in human cancer therapy. J Hematol Oncol. 2021;14(1):116. doi:10.1186/s13045-021-01127-w 34301278 PMC8299671

[26] Degirmenci U , Wang M , Hu J . Targeting aberrant RAS/RAF/MEK/ERK signaling for cancer therapy. Cell. 2020;9(1):198. doi:10.3390/cells9010198 PMC7017232 31941155

[27] Lukovic D , Moreno‐Manzano V , Lopez‐Mocholi E , et al. Complete rat spinal cord transection as a faithful model of spinal cord injury for translational cell transplantation. Sci Rep. 2015;5:9640. doi:10.1038/srep09640 25860664 PMC5381701

[28] Khankan RR , Griffis KG , Haggerty‐Skeans JR , et al. Olfactory ensheathing cell transplantation after a complete spinal cord transection mediates neuroprotective and immunomodulatory mechanisms to facilitate regeneration. J Neurosci. 2016;36(23):6269‐6286. doi:10.1523/JNEUROSCI.0085-16.2016 27277804 PMC4899528

[29] Anderson MA , O'Shea TM , Burda JE , et al. Required growth facilitators propel axon regeneration across complete spinal cord injury. Nature. 2018;561(7723):396‐400. doi:10.1038/s41586-018-0467-6 30158698 PMC6151128

[30] Petrović A , Ban J , Ivaničić M , Tomljanović I , Mladinic M . The role of ATF3 in neuronal differentiation and development of neuronal networks in opossum postnatal cortical cultures. Int J Mol Sci. 2022;23(9):4964. doi:10.3390/ijms23094964 35563354 PMC9100162

[31] Goshi N , Morgan RK , Lein PJ , Seker E . A primary neural cell culture model to study neuron, astrocyte, and microglia interactions in neuroinflammation. J Neuroinflammation. 2020;17(1):155. doi:10.1186/s12974-020-01819-z 32393376 PMC7216677

