# Supplementary material for: Serum response factor promoting axonal regeneration by activating the Ras–Raf‐Cofilin signaling pathway after the spinal cord injury
Source: CNS Neurosci Ther. 2024 Feb 8;30(2):e14585. doi: 10.1111/cns.14585 (PMC10851317; doi:10.1111/cns.14585)
Supplement: Supplementary file 1 — Table S1. [file CNS-30-e14585-s001.docx]

Supplemental Table 1. Primers for real-time quantitative PCR

| **Primer name Sequence (5'-3')** | |
| --- | --- |
| GAPDH |  |
| Forward: | GCACCGTCAAGGCTGAGAAC |
| Reverse: | TGGTGAAGACGCCAGTGGA |
| Ras |  |
| Forward: | TAGACACGAAACAGGCTCAGG |
| Reverse: | GGCATCGTCAACACCCTGTC |
| Raf |  |
| Forward: | CCCGTCCCGCTGAATACTAC |
| Reverse: | GGGTGGGGTAAGGGAACTTG |
| Cofilin |  |
| Forward: | TGCTACGAGGAGGTCAAGGA |
| Reverse: | CTGGAGGTGGCTCACAAAGG |
| SRF |  |
| Forward: | TAGACACGAAACAGGCTCAGG |
| Reverse: | GGCATCGTCAACACCCTGTC |
