# Supplementary figures and images for: Serum response factor promoting axonal regeneration by activating the Ras–Raf‐Cofilin signaling pathway after the spinal cord injury
Source: CNS Neurosci Ther. 2024 Feb 8;30(2):e14585. doi: 10.1111/cns.14585 (PMC10851317; doi:10.1111/cns.14585)

Fig.3-B

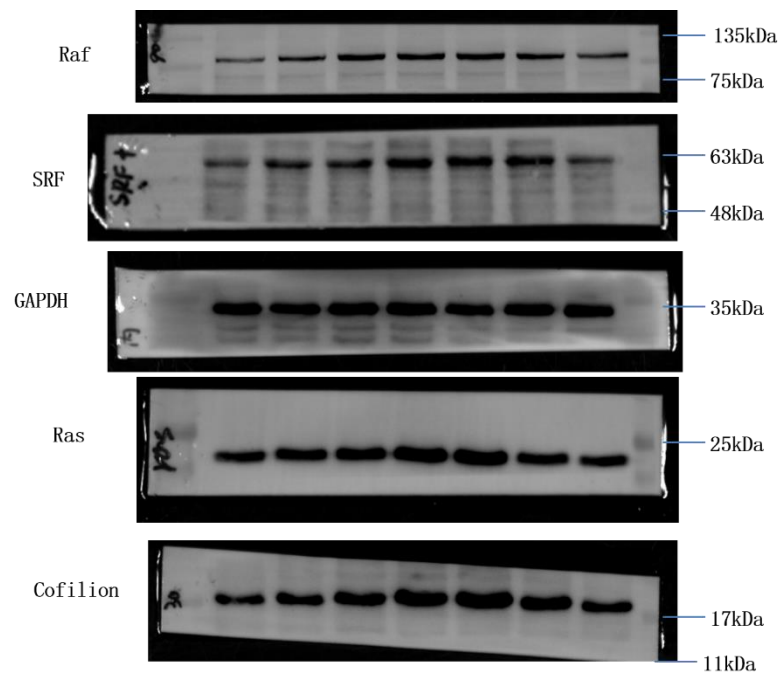

Fig.3-C

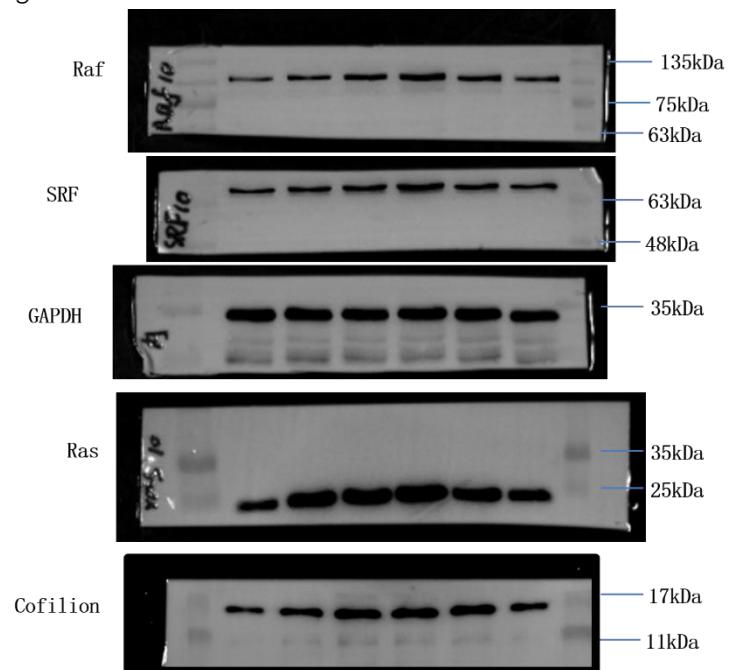

Figure.9-A

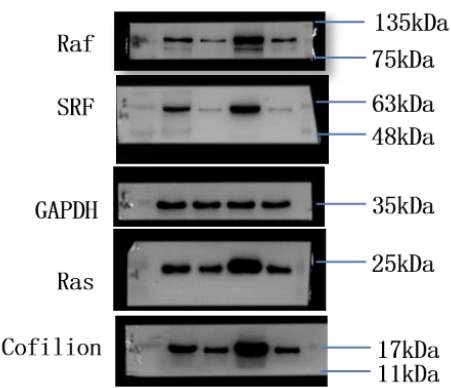

Figure.9-B

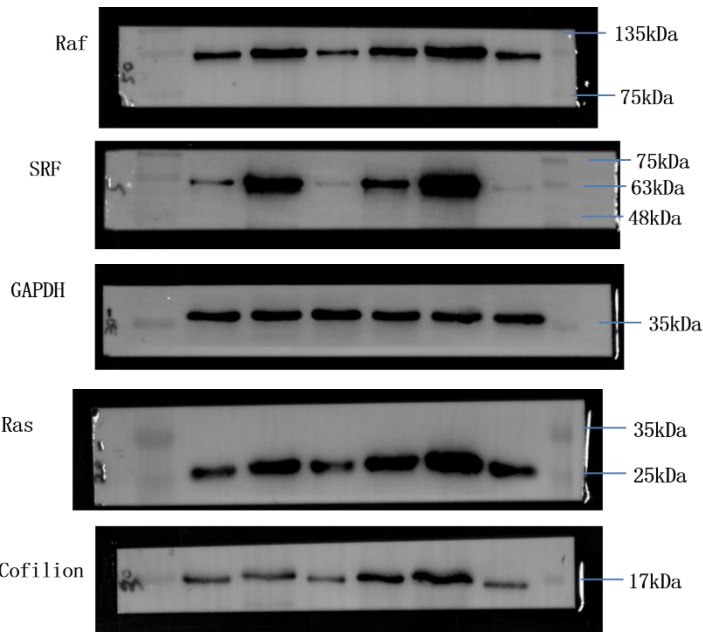

Supplement: Supplementary file 2 — Data S1. [file CNS-30-e14585-s002.pdf]
